# Anti-p200 Pemphigoid: A Systematic Review

**DOI:** 10.3389/fimmu.2019.02466

**Published:** 2019-10-22

**Authors:** Khalaf Kridin, A. Razzaque Ahmed

**Affiliations:** ^1^Department of Dermatology, Rambam Health Care Campus, Haifa, Israel; ^2^Department of Dermatology, Center for Blistering Diseases, Tufts University School of Medicine, Boston, MA, United States

**Keywords:** anti-p200 pemphigoid, anti-laminin gamma-1 pemphigoid, IIF of salt-split skin, immunoblotting, ELISA, BMZ autoantibodies, mucosal disease, psoriasis

## Abstract

The many clinical aspects of anti-p200 pemphigoid are not well-characterized. We aimed to analyze and correlate known existing data on the epidemiological, clinical, histological, and immunological features of anti-p200 pemphigoid. We performed a review using Medline, Embase, and Web of Science databases (1900–2018). Case reports and series of patients were included. A total of 68 eligible studies that comprised 113 anti-p200 pemphigoid patients were included in the qualitative analysis, where there was a mean age of onset of 65.5 years. All patients presented with bullae/vesicles, and 54.3% had urticarial plaques. A similarity to bullous pemphigoid was reported in 66.1% of cases, but palmoplantar (51.4%), cephalic (40.3%), and mucosal (38.5%) involvement, besides frequent development of scars/milia (15.7%), were reported. Autoantibodies against recombinant laminin γ1 were detected in the sera of 73.1% of patients. Psoriasis was present in 28.3% of anti-p200 pemphigoid patients, particularly among Japanese patients (56.4%). The incidence of pustular psoriasis in this subgroup, was significantly greater than in the normal population. In conclusion, the diagnosis of anti-p200 pemphigoid may be suspected when a subepidermal autoimmune blistering disease develops in a younger age group, along with significant acral and cephalic distribution and mucosal involvement.

## Introduction

Anti-p200 pemphigoid is a rare subepidermal autoimmune bullous disease (AIBD) initially described in 1996 (1, 2). This novel disease, presumed to be a subset of pemphigoid, was characterized by autoantibodies targeting a 200-kDa protein localized within the lower lamina lucida of the basement membrane zone (BMZ). Their sera bound to the dermal side of salt-split skin by indirect immunofluorescence (IIF) microscopy (3). Subsequent studies demonstrated that sera from 90% of anti-p200 pemphigoid patients recognized laminin γ1, which C-terminus was identified as an immunodominant region and utilized for immunoblotting and ELISA for diagnosis (4, 5). In outstanding research by German and Japanese investigators, using human foreskin and various mouse strains, the investigators clearly demonstrated that autoantibodies in anti-p200 pemphigoid sera are pathogenic, but their pathogenicity is not entirely mediated by autoantibodies against laminin γ1. The specific or precise role for laminin γ1 in pathogenesis requires further study, as *ex vivo* and *in vivo* studies did not show evidence of a direct pathogenic role of anti-laminin γ1 antibodies, leaving the true molecular identity of the pathogenic 200 kDa autoantigen yet to be fully characterized (6, 7). To elaborate, in two different mouse animal models for anti-laminin γ1 pemphigoid, although murine IgG of the recombinant laminin γ1 C-terminus bound to the epidermal basement membrane zone in the passive transfer model, no obvious blister formation was seen (7). In an earlier *ex vivo* model of autoantibody-mediated leukocyte-dependent neutrophil activation, human and rabbit IgG from the C-terminus of laminin γ1 failed to attract neutrophils at the dermal-epidermal-junction and to induce dermal-epidermal separation (6).

The clinical presentation of anti-p200 is polymorphic and may mimic bullous pemphigoid (BP), mucous membrane pemphigoid (MMP), and other subepidermal AIBD (8). However, data on its morphological features and the clinical course are limited, primarily because of the small number of reported cases and the lack of a large cohort of patients studied in detail with long-term follow-up.

We did not focus on therapy because it was considered beyond the scope of this analysis due to lack of uniformity, cohesive information, defined protocol, and outcome data. The aim of the current study was to perform a review of the available epidemiological, clinical, histological, and immunopathological data and the major comorbidities in patients with anti-p200 pemphigoid. The purpose was to help clinicians recognize this newly described clinical entity. This could result in early therapy and better prognosis.

## Materials and Methods

### Data Collection

The literature review was conducted using Ovid-Medline (1946–present), Embase (1947–present), and Web of Science (1900–present) to identify eligible articles. Publications until August 9th 2018, were searched. The search strategies are detailed in Supplementary Table 1.

### Selection of Articles

All publications reporting on one or multiple cases of anti-p200 pemphigoid were included. All cases were defined by the authors of the respective publications as anti-p200 pemphigoid based on the following three mandatory criteria: (i) clinical profile suggestive of subepidermal AIBD; (ii) reactivity to the 200 kDa protein or to the recombinant C-terminus of laminin γ1 by immunoblot analysis; and (iii) exclusion of other subepidermal AIBDs. Additionally, at least one of the following two minor criteria was required to establish the diagnosis of anti-p200 pemphigoid: (i) subepidermal cleft on histology; (ii) and direct immunofluorescence (DIF), demonstrating linear deposition of IgG and/or C3. Publications lacking these criteria were excluded.

### Data Extraction

The following information was obtained when authors provided it: age at onset, sex, ethnicity, morphological features of the mucocutaneous manifestation and their anatomic distribution, histopathology, immunopathology, comorbidities, and triggering factors (if known). All statistical analysis was performed using SPSS software, version 23 (SPSS, Chicago, IL, USA).

## Results

After a full-text review, 68 articles fulfilled the inclusion criteria, thereby providing 113 patients from 15 different countries that were included in the qualitative synthesis. Between 1996 and 2018, 50 cases (44.2%) were reported from Japan (Table 1).

**Table 1:** Demographic characteristics of the patients reported with anti-p200 pemphigoid.

Male patients, *n* (%)	85 (75.2%)
**Age at diagnosis**	
Mean (±SD)^*^	65.5 (15.9)
Median (range)	69 (5-94)
Mean age of male patients (±SD)^*^	66.0 (14.0)
Mean age of female patients (±SD)^*^	63.4 (20.8)
**Ethnicity of reported patients, *n* (%)**	
Asian	57 (50.4%)
Caucasians	22 (19.5%)
Jews	1 (0.9%)
African American	1 (0.9%)
Not reported	32 (28.3%)
**Geographical distribution of reported cases, % (*n*)**	
Japan	50 (44.2%)
France	18 (15.9%)
Germany	13 (11.5%)
Netherlands	12 (10.6%)
USA	7 (6.2%)
India	3 (2.7)
Poland	2 (1.8%)
Spain	2 (1.8%)
China	1 (0.9%)
UK	1 (0.9%)
Israel	1 (0.9%)
Croatia	1 (0.9%)
Greece	1 (0.9%)
Austria	1 (0.9%)
Korea	1 (0.9%)

*n, number; SD, standard deviation; USA, United States of America; UK, United Kingdom*.

* *Excluding the study of Meijer et al. (3) which did not report standard deviation*.

### Clinical Characteristics

The mean age at onset was 65.5 years (range 5–94) (9, 10). The majority of the patients were males (*n* = 85; 75.2%) and of Asian ancestry (*n* = 57, 50.4%; Table 1).

The clinical presentation was described as resembling other subepidermal AIBD and inflammatory dermatoses by authors in 68 (60.2%) patients. The leading similar condition was BP (*n* = 45; 66.2%), followed by linear IgA bullous dermatosis (LABD; *n* = 5; 7.4%) (9, 11–14), epidermolysis bullosa acquisita (EBA; *n* = 3; 4.4%) (15–17), dermatitis herpetiformis (DH; *n* = 3; 4.4%) (18–20), mucous membrane pemphigoid (*n* = 3; 4.4%) (21–23), and others (Table 2). In the remaining 45 patients, a similarity to a distinct clinical entity was not mentioned.

**Table 2:** Clinical and morphological characteristics of the reported patients with anti-p200 pemphigoid.

		**Number of reported cases**
**Similar condition**, ***n*** **(%)-reported in 68 cases**		
Bullous pemphigoid	45 (66.2%)	68
Linear IgA bullous dermatosis	5 (7.4%)	68
Vesicular pemphigoid	4 (5.9%)	68
Epidermolysis bullosa acquisita	3 (4.4%)	68
Dermatitis herpetiformis	3 (4.4%)	68
Mucous membrane pemphigoid	3 (4.4%)	68
Erythema gyratum repens	2 (2.9%)	68
Pemphigus	1 (1.5%)	68
Pompholyx	1 (1.5%)	68
**Morphology of cutaneous lesions**, ***n*** **(%)**		
Bullae/vesicles	113 (100%)	113
Urticarial plaques	50 (54.3%)	92
Scars/milia	16 (15.7%)	102
**Anatomical distribution of cutaneous lesions**, ***n*** **(%)**		
Extremities	78 (95.1%)	82
Trunk	58 (70.7%)	82
Palms and soles	38 (51.4%)	74
Head and neck	29 (40.3%)	72
Mucosal involvement	37 (38.5%)	96
**Mucosal involvement**, ***n*** **(%)**		
One mucosal surface	29 (30.2%)	96
Two mucosal surfaces concomitantly	6 (6.3%)	96
Three mucosal surfaces concomitantly	2 (2.1%)	96
Oral mucosa	31 (32.3%)	96
Anogenital mucosa	12 (12.5%)	96
Conjunctival mucosa	2 (2.1%)	96

*n, number. The right column represents the number of cases where the variable was reported or could be concluded. The ratios represent number of positive cases out of the number of reported cases*.

All patients presented with bullae and/or vesicles, 54.3% (50/92) had urticarial plaques, 15.7% (16/102) had scars and/or milia (Table 2).

The distribution of cutaneous lesions was reported in 72 patients. The extremities were the most frequently involved (95.1%), the trunk in 70.7%, and palmoplantar and cephalic involvement in 51.4 and 40.3% of patients, respectively. Generalized involvement was reported in 41.5% of patients. Mucosal involvement was reported in 38.5% of patients, with the oral mucosa (32.3%) being the most frequently involved. None of the patients had exclusively mucosal diseases (Table 2). Of great interest, the prevalence of mucosal involvement among Japanese patients (22.5%) was significantly lower when compared to the remaining patients (48.2%; *P* < 0.001).

### Histological Characteristics

Histology data was available on 100 (88.5%) patients. A subepidermal blistering was observed in all patients, along with mild to dense inflammatory infiltrates in the upper dermis composed of neutrophils and eosinophils in 41 (41.0%) and 15 (15.0%) patients, respectively. A mixed infiltrate of neutrophils and eosinophils was seen in 40 (40.0%) of patients. In two patients, an exclusively lymphocytic infiltrate was observed (24, 25); papillary microabscesses were observed in 11 (11.0%) patients, a predominantly neutrophilic infiltrate in 10 patients (2, 14, 18–20, 26–29) and a mixed infiltrate in one patient (3). Eosinophilic spongiosis was observed in two patients (3, 27) and ruptured secondary milia with inflammatory granulomatous in one patient (16).

### Immunopathological Characteristics

DIF microscopy of perilesional skin was reported in 98 (88.1%) patients. Deposition of IgG and C3 was observed in 77 (78.6%), whereas the simultaneous deposition of IgG, IgA, and C3 was observed in 10 (10.2%) patients (3, 9, 12, 14, 20, 30). Deposition of IgG or C3 alone was observed in two (2.0%) (16, 31) and four (4.1%) patients (22, 32–34), respectively. N-serration deposition pattern from a BMZ immunoreactant was shown in 11 of 13 (84.6%) patients, whereas this pattern was undetermined in the remaining two (15.4%) patients (3, 35, 36) (Table 3).

**Table 3:** Immunopathological and immunological characteristics of the reported patients with anti-p200 pemphigoid.

		**Number of reported cases**
**Direct immunofluorescence**, ***n*** **(%)**		
Linear deposition along the BMZ	113 (100%)	113
Linear deposition of IgG and C3	77 (78.6%)	98
Linear deposition of IgG, IgA, and C3	10 (10.2%)	98
Linear deposition of C3 alone	4 (4.1%)	98
Linear deposition of IgG, IgA, IgM, and C3	2 (2.0%)	98
Linear deposition of IgG alone	2 (2.0%)	98
N-serration deposition pattern	11 (84.6%)	13
U-serration deposition pattern	0 (0%)	13
Undetermined deposition pattern	2 (15.4%)	13
**NaCl-split indirect immunofluorescence deposition**, ***n*** **(%)**		
Dermal side	90 (83.3%)	108
Both dermal and epidermal sides (more prominently in the dermal side)	16 (14.8%)	108
Epidermal side	1 (0.9%)	108
Negative	1 (0.9%)	108
**Immunoblot analysis**, ***n*** **(%)**		
200-kDa dermal protein	111 (100%)	111
Recombinant C-terminus of laminin γ1	19 (73.1%)	26
**ELISA detecting recombinant monomeric C-terminal fragment of human laminin** **γ1**, ***n*** **(%)**		
	10 (100%)	10

*n, number; BMZ, basement membrane zone; Ig, immunoglobulin; ELISA, enzyme-linked immunosorbent assay. The right column represents the number of cases where the variable was reported or could be concluded. The ratios represent the number of positive cases out of the number of reported cases*.

On salt-split skin, binding to the dermal side of the split was reported in 90 patients (83.3%) and binding to both the dermal and epidermal sides in 16 patients (14.8%) (Table 3). Immunoblot analysis demonstrated reactivity to the 200 kDa protein in all 111 patients tested using human dermal extract (*n* = 108), epidermal extracts (*n* = 11) (10, 37) and keratinocytes extracts (*n* = 2) (32). Sera of several patients were tested on multiple extracts, and 10 of the 11 patients reacting to the epidermal extract were also seropositive on dermal extracts (10). In the remaining two patients, immunoblotting showed reactivity only to the recombinant C-terminus of laminin γ1 (38, 39). Of significant interest is that the sera of patients who were positive for antibodies when tested using immunoblot analysis with the recombinant C-terminus of laminin γ1 was positive in 17 of 24, with a 70.8% sensitivity (3, 9, 31, 38–43). Sera of 10 patients was tested by ELISA bound to a recombinant monomeric C-terminal fragment of human laminin γ1 (10, 44, 45) (Table 3).

Autoantibodies targeting other antigens were detected in 20 (17.7%). Autoantibodies against BP-180 alone, BP-230 alone and both BP-180 and BP-230 were identified in two (1.8%) (19, 46), four (3.5%) (2, 36, 47), and three (2.7%) patients (10, 48), respectively. Antibodies to Type-VII collagen were found in three (2.7%) patients (15, 16, 49), whereas autoantibodies against different subunits of laminin 332 were detected in 7 (6.2%) patients (21, 22, 34, 44, 50–52).

### Associated Comorbidities

An association with psoriasis was reported in 32 patients (28.3%). Psoriasis preceded the diagnosis of anti-p200 pemphigoid in all patients by a mean of 15.1 ± 10.4 years (median, 18 years; range, 0.1–30 years). Four (12.5%) patients had developed bullous disease with pustular psoriasis simultaneously (33, 35, 53, 54). The onset of anti-p200 pemphigoid was associated with erythrodermic psoriasis in one (3.1%) patient (55). The remaining patients (84.4%) had psoriasis vulgaris. Of interest, 28 (87.5%) patients with psoriasis were Japanese, whereas the remaining four (12.5%) were Europeans (10, 27, 56, 57).

Taken together, the prevalence of psoriasis in anti-p200 pemphigoid Japanese patients was 56.0% (95% CI, 42.3–68.8%) as compared to only 6.4% (95% CI, 2.5–15.2%) among non-Japanese patients (*P* < 0.001). There was a slightly higher frequency of males among anti-p200 pemphigoid patients with psoriasis compared to those without psoriasis (87.5 vs. 70.4%; *P* = 0.058). The age of presentation was comparable in the two subgroups (data not shown).

Concomitant malignancies were reported in three female patients. One had metastatic clear cell carcinoma of the ovary (21), one had uterine adenocarcinoma (58), and the other had metastatic esophageal adenocarcinoma (38). Other comorbidities reported included: end-stage kidney disease due to IgA nephropathy and glomerulonephritis (*n* = 2) (22, 34), ulcerative colitis (*n* = 1) (50), esophagitis (*n* = 1) (15), polyarteritis nodosa (*n* = 1) (59), and autosomal recessive congenital ichthyosis (*n* = 1) (36). Unproven trigger factors included scabies (*n* = 2) (60, 61) and use of penicillin (*n* = 1) (62).

## Discussion

This review highlights the multiple aspects of a recently described interesting subset of pemphigoid. Based on the current information, its main focus is the clinical features. Many of the unique characteristics and associations could be important to cutaneous biology and the pathogenesis of autoimmunity. A heterogeneous clinical profile that mimicked BP was present in the majority of patients, accompanied by a high prevalence of cephalic, palmoplantar and mucosal involvement. Autoantibodies against the recombinant C-terminus of laminin γ1 were detected by immunoblotting in 73.1% of patients. The prevalence of coexistent psoriasis was significantly higher than in the general population. Interestingly, the presence of psoriasis was very significantly higher in Japanese than non-Japanese patients (56.0 vs. 6.4%, respectively).

Palmoplantar lesions and mucosal involvement in BP has been reported in up to 20% of patients (63–65), and these regions were affected in 51.4 and 38.5% of patients with anti-p200 pemphigoid, respectively. Interestingly, scars and milia were reported in 3.4% of patients with BP (63) but in 15.7% of patients with anti-p200 pemphigoid. In the clinical criteria suggested by Vaillant et al. (66), which yield a positive predictive value of 95% for a clinical diagnosis of BP, it is the absence of each of the three aforementioned features that has been identified as an independent predictive criterion for the diagnosis of BP.

The age at onset of patients with anti-p200 pemphigoid (65.5 years) is younger than many with BP, where it commonly occurs in those older than 70 (67–69). An overwhelming preponderance of males (75.2%) were reported with anti-p200 pemphigoid, which contrasts with the female-to-male ratio ranges between 1.04 and 5.1 in different cohorts of BP (69).

In 2009, Dainichi et al. (4) found that 90% of sera tested from anti-p200 pemphigoid patients reacted to the C-terminus of recombinant laminin γ1, using an immunoblot assay. This observation has been confirmed by Groth et al. (5). This analysis demonstrates that sera from 73.1% of the patients reported reacting to the C-terminus of recombinant laminin γ1 protein using the immunoblotting assay. With respect to the ELISA using a monomeric C-terminal fragment of human laminin, the sensitivity was estimated at 68.6% (5).

The incidence of anti-p200 pemphigoid is considerably low amongst AIBD (70). In a cohort of 145 Japanese patients with various AIBDs, anti-p200 pemphigoid was the second most prevalent AIBD (37.2%). The prevalence of psoriasis among non-Japanese anti-p200 pemphigoid patients (6.4%) was greater than its incidence in the general population of the world (71, 72). The pathomechanism of this ethnic association is yet to be established (70). Pustular psoriasis was present in 12.5% of p-200 pemphigoid patients. This incidence is significantly higher than the 1.3% reported in a cohort of 104,669 patients with different variants of psoriasis (*P* < 0.001) (73). This observation confirmed an earlier Japanese study which reported that 53.8% of patients with pustular psoriasis and AIBD had anti-p200 pemphigoid (70). The molecular basis for this rare and unique combination of clinical entities has not been clearly defined and warrants attention. Senescence was suggested to alter the distribution and amount of proteins in the BMZ, thus increasing its antigenicity and raising the risk of the development of an autoimmune response against its components (74). Since the cell cycle and turnover of the epidermal keratinocytes are extremely accelerated in psoriasis, it was recently assumed that the extracellular matrix in psoriatic skin may simulate the senescent extracellular matrix and contribute to the development of BP and other AIBDs that were found to be associated with psoriasis (75).

Our current knowledge of anti-p-200 pemphigoid brings into focus several important and interesting issues. Traditionally, pemphigoid is divided into BP and mucous membrane pemphigoid. It is possible that because anti-p200 pemphigoid has certain features of both conditions, it could be the bridge or missing link between them. Some anti-p200 pemphigoid also has features of other AIBD, since their sera contain autoantibodies associated with them. It would be interesting to speculate whether this was due to epitope spreading (76). Patients with systemic lupus erythematosus have multiple pathogenic autoantibodies per patient (77), including multiple phenotypes and symptomatologies.

An additional unsolved question is whether anti-p-200 pemphigoid is truly rare or whether it is rather underdiagnosed worldwide. In the US, tests for detecting autoantibodies to laminin γ1 are commercially unavailable, though they are available in Germany and Japan. Moreover, investigators in those countries, whose laboratories are equipped to perform these assays, provide them to their colleagues. The majority of patients are thus reported from Japan and Europe. Unless there are very specific unidentified reasons, there are generally no reasons to believe that it cannot not be as prevalent in other countries. Unless more patients are studied, serologically characterized and followed up for several years, this challenging variant of pemphigoid will remain less well-studied and understood. This would be tragic, because it could help advance our knowledge.

The main limitations of this review are that it was based on case reports and small case series and that it is retrospective. Some of the variables were not provided by some authors. Data on treatment were scarce, limited and not comprehensive. Limited follow-up did not provide a clear clinical course or an overview of the impact of treatment on clinical course. The universal lack of the ELISAs makes it impossible to assess its global presence. Since psoriasis is present universally, its relationship could not be accurately assessed.

In conclusion, this new subset provides, to clinicians and scientists alike, a unique opportunity to study several new frontiers of cutaneous biology, inflammation, immunology, scar formation and disease pathogenesis. The location of laminin γ1 provides the opportunity to study interactions amongst other BMZ proteins. An association with psoriasis provides a playground to study what influences neutrophils to cause different pathologies and clinical profiles. The presence of scarring in mucosal tissues of some patients and not in others could provide important leads into what thrusts patients into one direction or the other. This opportunity, provided by nature, should not be missed but utilized to the advantage of our patients and the progress of our specialty.

## Author Contributions

AA and KK contributed to the concept, content, and writing of the manuscript.

### Conflict of Interest

The authors declare that the research was conducted in the absence of any commercial or financial relationships that could be construed as a potential conflict of interest.
