# Supplementary material for: Anti-p200 Pemphigoid: A Systematic Review
Source: Front Immunol. 2019 Oct 22;10:2466. doi: 10.3389/fimmu.2019.02466 (PMC6817563; doi:10.3389/fimmu.2019.02466)
Supplement: Supplementary file 1 [file Table_1.docx]

**Supplementary table 1-** Literature search strategy for each one the databases examined

| **Literature search scheme for OVID Medline (97 results)**  **Search String:** |
| --- |
| \| #11 \| Search (#9 OR #10) \| \| --- \| --- \| \| #10 \| Search dermal 200 kD antigen \| \| #9 \| Search (#1 AND #8) \| \| #8 \| Search (#2 OR #3 OR #4 OR #5 OR #6 OR #7) \| \| #7 \| Search ((LAMC1-protein[Text Word] OR LAMC1 protein[Text Word]) OR LAMC1) \| \| #6 \| Search ((Anti-laminin gamma1) OR (Antilaminin gamma1) OR Antilaminin-gamma1 OR Anti-laminin-gamma1 OR "Anti-laminin-gamma 1") OR (Anti-laminin "gamma 1") OR (Antilaminin "gamma 1") OR (Antilaminin gamma-1) OR (Antil-aminin gamma-1)) \| \| #5 \| Search ("laminin gamma1"[Text Word] OR "laminin-gamma1"[Text Word] OR "laminin gamma-1"[Text Word] OR "laminin gamma 1"[Text Word] OR "laminin-gamma 1"[Text Word] OR laminin-gamma-1 OR) \| \| #4 \| Search laminin gamma 1[Supplementary Concept] \| \| #3 \| Search (Anti-p200[Text Word] OR "Anti-p 200"[Text Word]) \| \| #2 \| Search (p-200[Text Word] OR p200[Text Word] OR "p 200"[Text Word]) \| \| #1 \| Search pemphigoid[Title/Abstract] \| |
| **Literature search scheme for Embase (134 results)**  **Search String:** |
| \| #3 OR #9 \| #10 \| \| --- \| --- \| \| #1 AND #8 \| #9 \| \| #2 OR #4 OR #5 OR #6 OR #7 \| #8 \| \| 'lamc1':ab,ti \| #7 \| \| ((laminin NEAR/1 γ1):ab,ti) OR (('anti laminin' NEAR/1 γ1):ab,ti) OR ((antilaminin NEAR/1 γ1):ab,ti) OR ((laminin NEAR/1 'γ 1'):ab,ti) OR ((antilaminin NEAR/1 'γ 1'):ab,ti) OR (('anti laminin' NEAR/1 'γ 1'):ab,ti) \| #6 \| \| (('anti laminin' NEAR/1 gamma1):ab,ti) OR ((antilaminin NEAR/1 gamma1):ab,ti) OR (('anti laminin' NEAR/1 'gamma 1'):ab,ti) OR ((antilaminin NEAR/1 'gamma 1'):ab,ti) \| #5 \| \| ((laminin NEAR/1 'gamma 1'):ab,ti) OR ((laminin NEAR/1 gamma1):ab,ti) \| #4 \| \| 200 AND kd AND dermal AND antigen \| #3 \| \| 'p 200':ab,ti OR p200:ab,ti OR 'anti p200':ab,ti OR 'anti p 200':ab,ti \| #2 \| \| pemphigoid:ab,ti \| #1 \| |
| **Literature search scheme for Web of Science (99 results)**  **Search String:** |
| \| #6 OR #5 OR #4 OR #3 OR #2 OR #1 \| # 7 \| \| --- \| --- \| \| Indexes=SCI-EXPANDED, SSCI, A&HCI, ESCI Timespan=All years \|  \| \|  \|    \|  \| \| --- \| \| \| TI=(("Anti-laminin gamma1" OR "antilaminin gamma1" OR "Antilaminin-gamma1" OR "Anti-laminin-gamma1" OR "Anti-laminin-gamma 1" OR "Anti-laminin gamma 1" OR "Antilaminin-gamma 1" OR "Antilaminin gamma 1" OR "anti-laminin-gamma-1" OR "Antilaminin-gamma-1" OR "Antilaminin gamma-1") NEAR/2 Pemphigoid) OR (TS=(("Anti-laminin gamma1" OR "antilaminin gamma1" OR "Antilaminin-gamma1" OR "Anti-laminin-gamma1" OR "Anti-laminin-gamma 1" OR "Anti-laminin gamma 1" OR "Antilaminin-gamma 1" OR "Antilaminin gamma 1" OR "anti-laminin-gamma-1" OR "Antilaminin-gamma-1" OR "Antilaminin gamma-1") NEAR/2 Pemphigoid)) \| # 6 \| \| Indexes=SCI-EXPANDED, SSCI, A&HCI, ESCI Timespan=All years \|  \| \|  \|    \|  \| \| --- \| \| \| TI=(("laminin gamma1" OR "Laminin-gamma1 " OR "laminin gamma-1" OR "laminin gamma 1" OR "Laminin-gamma 1" OR "Laminin-gamma-1") NEAR/2 pemphigoid) OR TS=(("laminin gamma1" OR "Laminin-gamma1 " OR "laminin gamma-1" OR "laminin gamma 1" OR "Laminin-gamma 1" OR "Laminin-gamma-1") NEAR/2 pemphigoid) \| # 5 \| \| Indexes=SCI-EXPANDED, SSCI, A&HCI, ESCI Timespan=All years \|  \| \|  \|    \|  \| \| --- \| \| \| TI=(laminin NEAR/4 gamma-1 NEAR/4 pemphigoid) OR TS=(laminin NEAR/4 gamma-1 NEAR/4 pemphigoid) \| # 4 \| \| Indexes=SCI-EXPANDED, SSCI, A&HCI, ESCI Timespan=All years \|  \| \|  \|    \|  \| \| --- \| \| \| TI=((200-kD dermal antigen) OR (200 kd dermal antigen) OR (dermal 200-kD antigen) OR (dermal 200 kD antigen) OR (200-kD dermal-antigen) OR (200 kD dermal-antigen)) OR TS=((200-kD dermal antigen) OR (200 kd dermal antigen) OR (dermal 200-kD antigen) OR (dermal 200 kD antigen) OR (200-kD dermal-antigen) OR (200 kD dermal-antigen)) \| # 3 \| \| Indexes=SCI-EXPANDED, SSCI, A&HCI, ESCI Timespan=All years \|  \| \|  \|    \|  \| \| --- \| \| \| TI=((Anti-p200 OR "Anti p200" OR "Anti p-200" OR "Anti-p 200" OR Anti-p-200) NEAR/2 pemphigoid) OR TS=((Anti-p200 OR "Anti p200" OR "Anti p-200" OR "Anti-p 200" OR Anti-p-200) NEAR/2 pemphigoid) \| # 2 \| \| Indexes=SCI-EXPANDED, SSCI, A&HCI, ESCI Timespan=All years \|  \| \|  \|    \|  \| \| --- \| \| \| TI=((p200 OR "p 200" OR p-200) NEAR/2 pemphigoid) OR TS=((p200 OR "p 200" OR p-200) NEAR/2 pemphigoid) \| # 1 \| |
